# Supplementary material for: Interrogating the Diversity of Vaginal, Endometrial, and Fecal Microbiomes in Healthy and Metritis Dairy Cattle
Source: Animals (Basel). 2023 Mar 31;13(7):1221. doi: 10.3390/ani13071221 (PMC10093325; doi:10.3390/ani13071221)
Supplement: Supplementary file 1 [file animals-13-01221-s001.zip › Supplementary Figures_TT.pdf]

Supplementary Figure S1

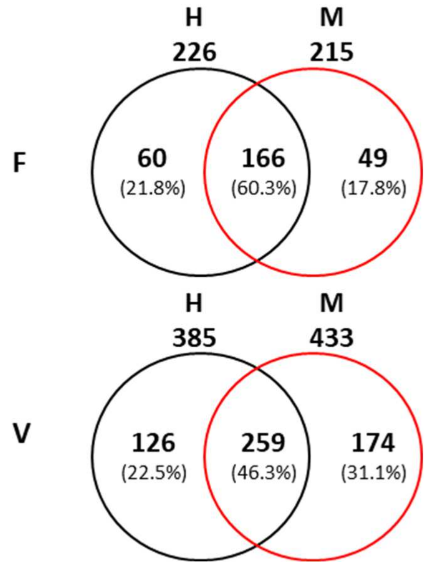

Supplementary Figure S1: Venn diagrams showing the overall distribution of genus OTUs between pre-calving fecal and vaginal microbiomes of the metritis and healthy cows. OTUs found in at least one cow per group were included in the comparison. Fecal microbiomes shared more (60.3% vs 46.3%) OTUs than vaginal microbiomes between healthy and metritis cows.

Supplementary Figure S2

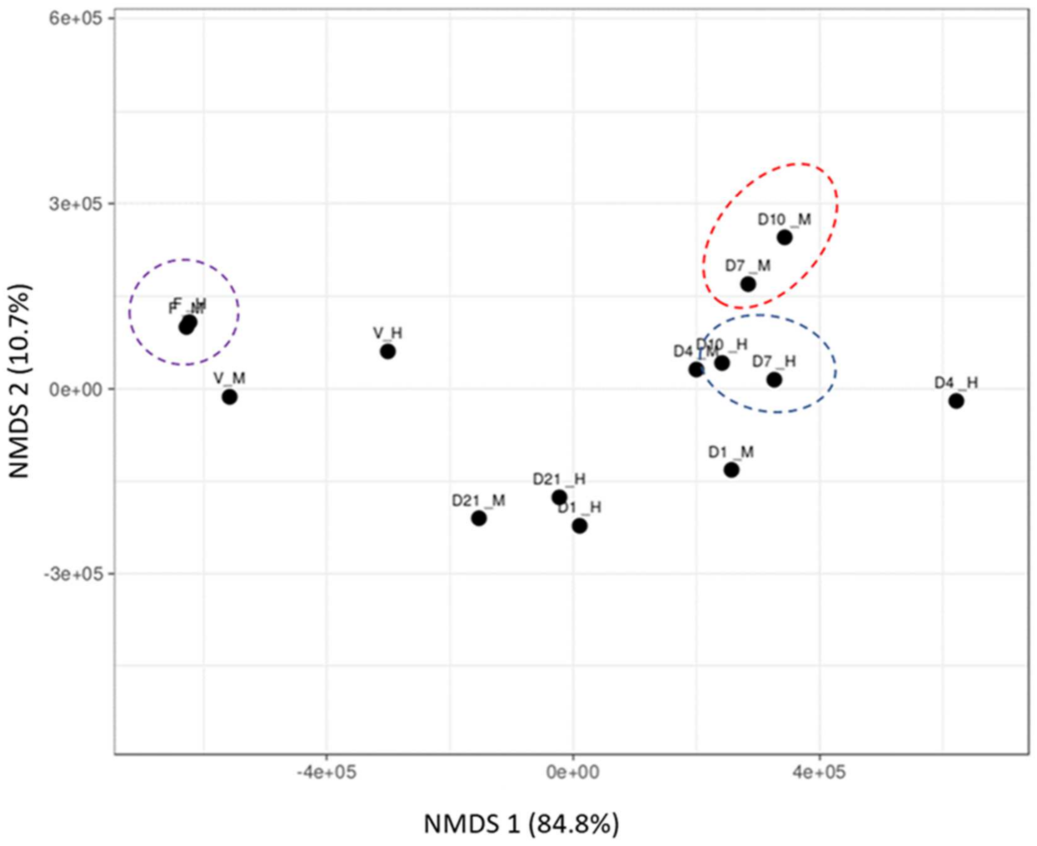

Supplementary Figure S2: NMDS plots showing compositional similarity between the metritis and healthy cow groups based on Bray-Curtis similarity distances derived from the averaged taxonomic compositions (taxa presence and absence and relative abundances) between the metritis and healthy cow groups.

Supplementary Figure S3

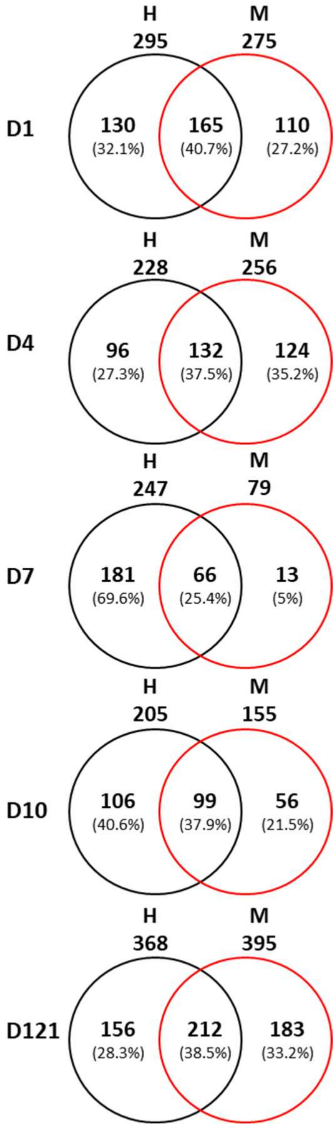

Supplementary Figure S3: Venn diagrams showing the distribution of low abundance genus OTUs between the metritis and healthy cow postpartum uterine microbiomes. OTUs present in at least one cow per group is shown. The healthy and Metritis postpartum uterine communities are most distinctive at D7 and D10. At these time points the metritis and healthy core microbiomes have the smallest number of genera in common.
